# Investigation of ^3^H, ^99^Tc, and ^90^Sr transport in fractured rock and the effects of fracture-filling/coating material at LILW disposal facility

**DOI:** 10.1007/s10653-018-0123-y

**Published:** 2018-05-23

**Authors:** Won-Seok Kim, Sangsoo Han, Jinmo Ahn, Wooyong Um

**Affiliations:** 1 Division of Advanced Nuclear Engineering (DANE), Pohang University of Science and Technology (POSTECH), 77 Cheongam-ro, Nam-gu, Pohang, Gyungbuk 790-784 South Korea; 2 Division of Environmental Science and Engineering (DESE), Pohang University of Science and Technology (POSTECH), 77 Cheongam-ro, Pohang, South Korea

**Keywords:** Radionuclide transport, Repository, Fractured rock, Unsaturated zone, Adsorption

## Abstract

Batch adsorption, batch diffusion, and flow-through column experiments were conducted using groundwater and fractured rock collected in unsaturated zone to increase our understanding of sorption and transport behavior of radionuclides. Increasing *K*_d_ values were observed in the sequence ^90^Sr, ^99^Tc, and ^3^H regardless of the geological media tested. For all sorbing radionuclides, *K*_d_ values for the fracture-filling/coating material were observed to be higher than those for without fracture-filling/coating material regardless of the groundwater. These higher *K*_d_ values are the result of zeolite mineral in filling/coating material of fractured rock. The batch diffusion and flow-through column experiments were also conducted using the same fractured rock sample, and the results of diffusion and column experiments showed similar trend of radionuclide sorption and transport to sorption experiment. In this study, sorption *K*_d_ of radionuclide was determined and used to increase our understanding of radionuclide retardation through fracture-filling/coating materials.

## Introduction

A final repository for the radioactive waste is required in South Korea. The low- and intermediate-level radioactive waste (LILW) repository which is a rock cavern type was constructed and opened in Wolsong, Gyeongju, South Korea. In addition, there is surface type repository is planned to construct at same site in Gyeongju. The Wolsong LILW disposal facility is the world’s first repository to employ both rock cavern and surface repositories on the same site. With the development of nuclear energy, large amounts of radioactive wastes have been released into the natural environment (Chapman et al. 2012; Linsley 1989). The radionuclide can be released from the drums after long storage periods and can simultaneously penetrate through artificial barrier from the near surface repository. It can be transported by the flow of rainfall, exist with pore water through fractures in unsaturated zone, and is reached to groundwater flow. Released radionuclides to vadose zone and ultimately groundwater can be transported by groundwater flow through porous materials or fractures (Mann et al. 2001).

The potential release and mobility of radionuclides from LILW disposal facility may pose environmental problems to adjacent areas. The presence of released radionuclides can affect human health (Chapman et al. 2012; Linsley 1989). The transport of chemical compounds in porous geological media cannot be quantified adequately in natural groundwater systems. When the particles move within flowing water, there are advection, dispersion, and diffusion. The term advection refers to the transport of a solute by the bulk movement of groundwater. Dispersion is caused by the different flow paths water particles take in a geological medium. Molecular diffusion is caused by random molecular motion due to the thermal kinetic energy of the solute (Schulze-Makuch 2009). The physical and chemical processes of advection, dispersion, diffusion, sorption, and degradation should be discussed and have to be considered when evaluating the transport of chemical contaminant compounds and radioactive compound in the subsurface. These processes affect the transported substance as well as the transport medium (rocks or sediments in the subsurface) and result in complex distributions of the substance in natural groundwater systems. Because retardation phenomena such as sorption and precipitation are one of the important processes for retarding radionuclide migration when radionuclides are released from a radioactive waste repository, an understanding of sorption and transport behavior of radionuclides is required (Neretnieks et al. 1980). In order to complete a safety assessment of a radioactive waste repository, it is necessary to obtain sorption data for radionuclides. Although some batch sorption experiments were previously conducted for radionuclides with various waste facility sediments and waste types, there is no research about transport under unsaturated condition.

Rainwater flow occurs through the pore spaces in geological medium which is called a porous medium. In some geological media, groundwater flow occurs through fractures. When evaluating solute transport in fractured media, future investigation has to be conducted in regard to fluid flow and transport. When released radionuclides from LILW repository migrate through fractured rock in unsaturated zone, they also diffuse into the filling/coating material adjacent to fractures and are retarded by sorption onto filling/coating material in the fracture surface (Baik et al. 2008). Therefore, it is important to investigate the sorption characteristics of radionuclide and the relationship between transport behavior (retardation) of radionuclides through fractured rock and fractured filling/coating material which is existed on surface of fractured rock in unsaturated zone for the safety assessment and long-term performance of repository. The long-term risks predicted for a waste disposal facility are affected by the potential mobility of high-risk radionuclides that are expected to be highly mobile.

**Fig. 1:**
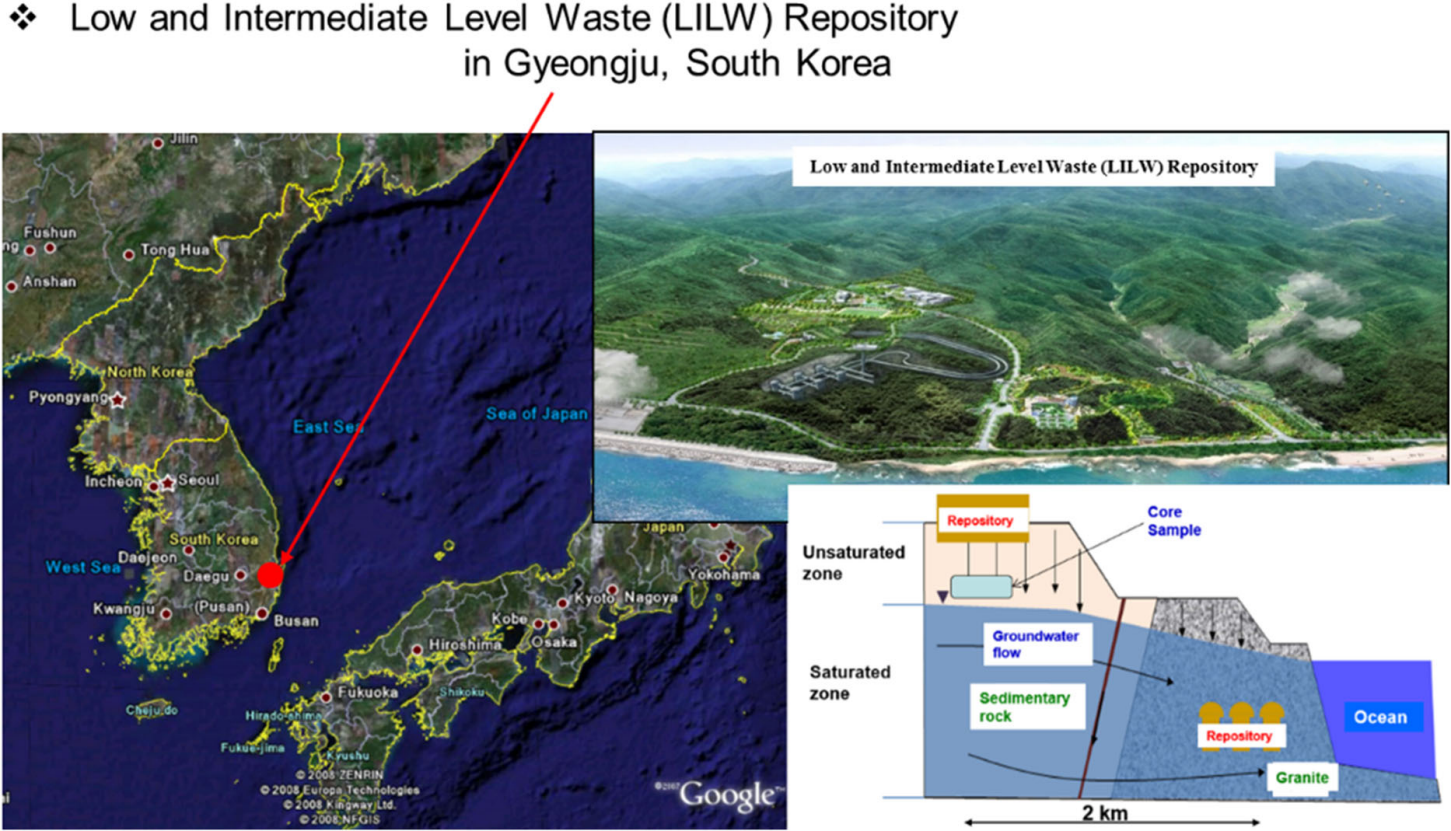
The in situ core sampling point at LILW repository in Gyeongju, South Korea

To determine the relationship between the sorption of radionuclide and site characteristics which fracture-filling/coating material, batch sorption experiment for radionuclides such as ^3^H, ^99^Tc, and ^90^Sr were conducted using groundwater and solid materials from the LILW repository site. The map of sampling point is shown in Fig. 1. In this study, the ^3^H, ^99^Tc, and ^90^Sr were used because they are a health hazard radioactive material which causes the cancer induction from beta particles associated with its radioactive decay and very common in nuclear test sites. Radioactive tritium (^3^H) has a half-life (*t*_½_) of 12.32 years. Tritium is radiological hazardous, can be incorporated into humans through respiration, ingestion, and diffusion through skin, and can be taken up by all hydrogen-containing molecules, distributing widely on a global scale (Okada et al. 1993). Radioactive strontium (^90^Sr [II]) with a half-life of 28 years is an important product of the nuclear reactor operations, nuclear weapons testing, and reactor accidents (Riley et al. 1992). Technitium-99 (^99^Tc) is a long-lived (*t*_½_ = 2.13 × 10^5^ years) pure β-emitter (*E*_βmax_ = 292 keV) and is produced as a fission product of uranium-235 and high water solubility and mobility in aquatic ecosystems under oxidizing environments (Wildung et al. 1979; Schulte et al. 1987).

The objective of this work is to investigate the sorption of radionuclides including measurements of the *K*_d_ values of ^3^H, ^99^Tc, and ^90^Sr for fracture-filling/coating material, and the transport to radionuclides released to the vadose zone at the Wolsong LILW repository in unsaturated zone. In this study, batch sorption, batch diffusion, and flow-through column experiments were conducted with LILW repository aquifer materials which unsaturated zone to increase our understanding of sorption and transport behavior of radionuclides.

## Materials and methods

### Radionuclides, groundwater, and solid materials

In the experiment, T_2_O, NH_4_TcO, and SrI were used. The groundwater was sampled near the LILW repository site at June 11, 2015, and the temperature is 17.5 °C, pH is 6.8, EC is 201.7 μS/cm, and DO is 4.5 mg/L. Fractured rock samples were also collected near the LILW repository site at unsaturated zone, and fractured rock was characterized by X-ray microtomography (XMT, X-Tek/Metris XTH 320/225 kV, UK) analysis and fractured filling/coating material was investigated by X-ray diffraction (XRD, Rigaku, D/MAX-2500/PC, Japan), scanning electron microscopy (SEM, JEOL, JSM-7401F, Japan), and polarized light microscopy (PLM, AmScope, PZ620T-MF603, USA). In this work, liquid scintillation analyzer (PerkinElmer 3100TR) was used to analyze the transport behavior of ^3^H and ^90^Sr, and inductively coupled plasma–mass spectrometry (PerkinElmer, NexION 300D) was used for ^99^Tc. The Br concentrations in the effluents were determined using an ion chromatography (IC, ICS-2100, Thermo) and used to develop Br breakthrough curves.

### Batch sorption experiments

**Fig. 2:**
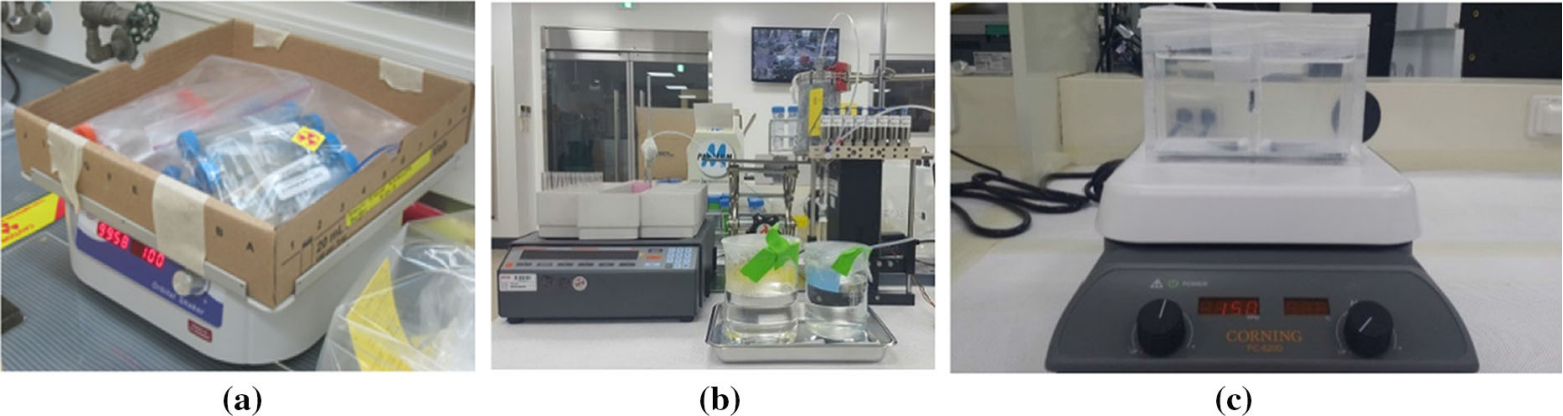
Experiment for **a** batch sorption, **b** flow-through column, and **c** diffusion

Solid material (1.0 g for ^3^H, 1.0 g for ^99^Tc, 0.5 g for ^90^Sr) was used in 10 mL of groundwater for the batch sorption experiments. Figure 2a shows the batch sorption experiment equipment. Solid material is prepared for comparison of radionuclide adsorption to fracture-filling/coating material. It is fractured rock with fracture-filling/coating materials and without fracture-filling/coating materials. The solid sample was sieved and the 75–250 μm size of solid material was used for batch sorption experiments. Solid material was pre-equilibrated through contact with a sufficient amount of groundwater for 1 day for the sorption experiments. Pre-equilibrated step was repeated until the pH of the solution was maintain with the pH of groundwater (pH = 6.8). After pre-equilibration, control batch test which without radionuclide was performed to determine the initial concentrations of the radionuclides and the mass loss by sorption onto bottle walls. The radionuclide was spiked into the pre-equilibrated reacting bottles to attain the required radionuclide concentration (^3^H: 100 Bq/mL, ^99^Tc: 100 Bq/mL, ^90^Sr: 20 Bq/mL). Solid material was shaken at 80 rpm with radionuclide spiked groundwater for 14 days. The pH was adjusted to maintain the initial pH of the groundwater by 0.1 M HNO_3_ or NaOH solution during experiment. All experiments were conducted at room temperature using reagent grade chemicals. A blank sample, which contained no solid material, was prepared to determine the initial activity of each radionuclide. After finish the batch sorption experiment, the solution was separated by using a syringe filter of 0.45 μm pore size (Whatman) and stored to determine the concentration of radionuclides. All batch sorption experiments were carried out in triplicate.

### Distribution coefficients

Solid/liquid partition coefficients (K_d_), generally referred to as a distribution coefficient are commonly used to reveal the mobility and distribution of elements in the environment (Eil-Garcia et al. 2009a, b; Vandenhove et al. 2009). In this study, *K*_d_ is used to reveal the sorption capacity and the relation between the mobility of radionuclides and elements of concern from LILW repository site. The *K*_d_ is the ratio of the concentration of radionuclide on a fractured rock divided by the equilibrium concentration in the contacting liquid phase (water). *K*_d_ values are highly dependent on environmental factors, including the performance assessment of a radioactive waste repository but not limited to the amount and type of minerals, the solution composition, ionic strength, Eh pH, redox condition, particle size distribution, organic matter content, biological activity and temperature.

A distribution coefficient, *K*_d_ (m^3^/kg), defined as:

1 $${K_{\text{d}}} = \frac{{\left( {{C_0} - \;{C_{\text{q}}}} \right)}}{{{C_{\text{q}}}}}\;\frac{V}{M}$$

where *C*_q_ is the measured concentration of the radionuclide in the solution phase (Bq/mL), C_0_ is the total concentration of the radionuclide (Bq/mL) initially added to the solution, *V* is the solution volume in contact with the solid (m^3^), and *M* is the amount of solid (kg).

### Flow-through column experiment

**Fig. 3:**
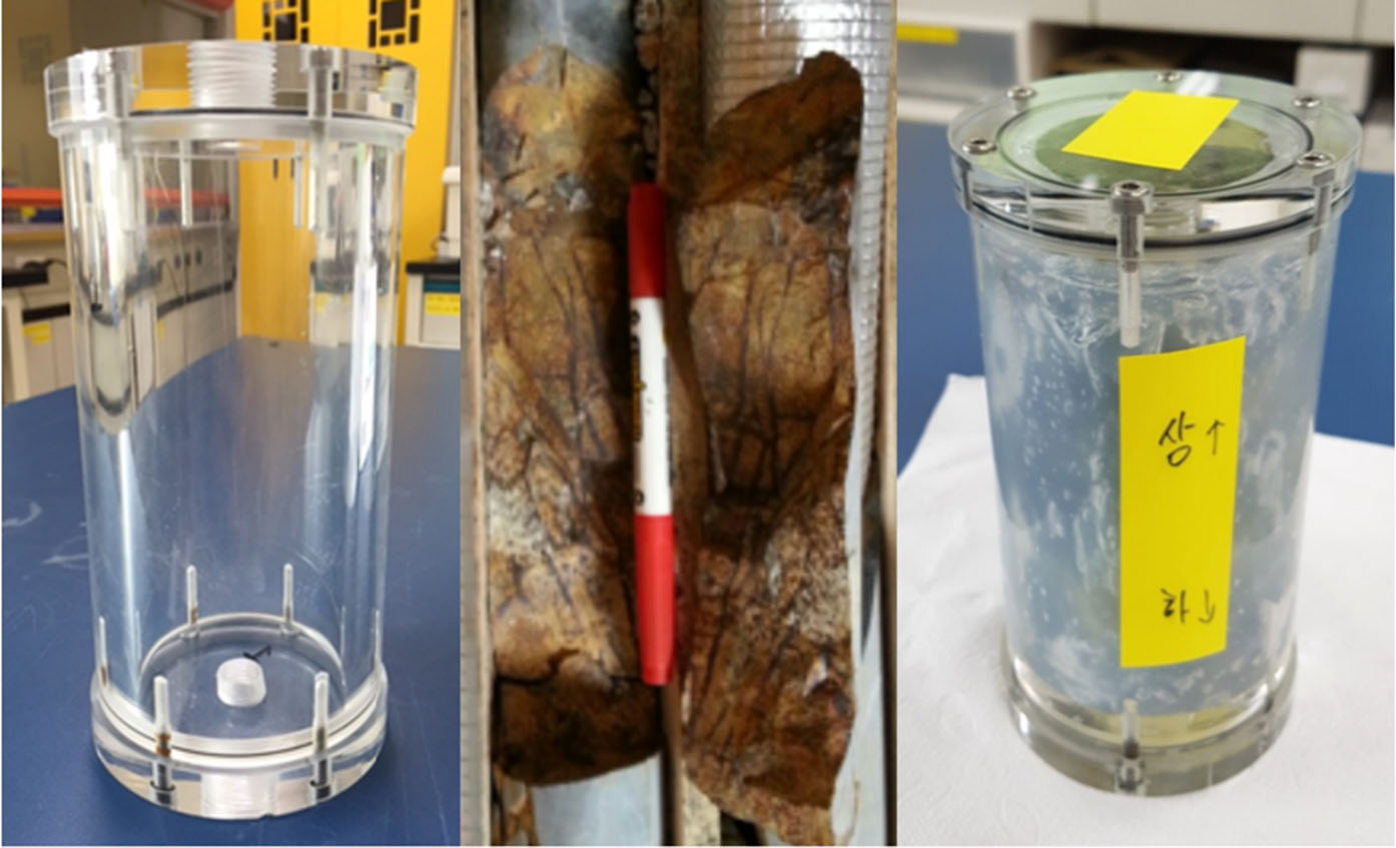
Fractured rock sample and column

The flow-through column experiment was conducted using a polyether ether ketone (PEEK) column (6 cm inner diameter and 13.5 cm height). The flow-through column experiment equipment is shown in Fig. 2b. Fractured rock sample (5 cm diameter and 12.5 cm height) was installed inside of column. The column and fractured rock sample is shown in Fig. 3. Each end of the column and the space between the inner wall of column and outside of fractured rock sample was packing using silicon in order to prevent the water flow through the space. The dry column weight and saturated column by deionized water (DIW) weight were measured to make unsaturated condition of column experiment. The column experiment was performed under unsaturated flow conditions, in order to investigate the transport of radionuclide in disposal site which situated in unsaturated moisture conditions. DIW was flowed through saturated column to stabilize over a 2-day period, and moisture contents were adjusted and monitored by RCON2™ (Giatec Scientific Inc.) resistor to 30–35%. Constant flow rate was maintained with a syringe pump (Kloehn) and downward flow was used to maintain unsaturated column condition.

Bromide in deionized water was introduced as a non-reactive conservative tracer to characterize the hydrodynamic properties of column and determine the dispersion coefficient. After obtaining Br breakthrough for column, the Br input solution was immediately replaced by the same background solution. The influent was switched to groundwater spiked with individual radionuclides (^3^H, 500 Bq/mL; ^99^Tc, 500 Bq/mL). respectively, and stable strontium (10^−1^ M). After obtaining each radionuclides breakthrough curves (BTCs), the input solution was immediately replaced by the same background solution in each step. Effluent samples were collected with a fraction collector (CF-2, Spectra Chrom), filtered through 0.45-μm pore-size filters. BTCs were graphically represented by plotting the relative activity, C/C_0_, versus pore volumes eluted. A pore volume was defined as the volume of water retained within the pore space of the fractured rock sample and measured by the weight difference before and after column saturation.

The transport parameters were determined numerically by the model fit using the measured BTCs with the CXTFIT code (Parker et al. 1984; Toride et al. 1995). The BTC of the non-reactive conservative tracer (Br) in column was first fitted to determine the hydrodynamic dispersion coefficient (*D*) of column. The obtained *D* value was used as a fixed parameter to obtain the retardation factor (*R*) for Br fixed at 1.0 for ^3^H, ^99^Tc and strontium transport using same equilibrium model fit. Retardation factor, *R* is defined as

2 $$R = 1 + {\text{\;}}\frac{{\left( {{K_{\text{d}}} \cdot {\rho_{\text{b}}}} \right)}}{\theta }$$

where *K*_d_ (mL/g) is sorption distribution coefficient, *ρ*_b_ (g/cm^3^) is bulk density, and *θ* is porosity of the packed column. Because the half-lives for 3H (*t*_1/2_ = 12.3 years), ^99^Tc (*t*_1/2_ = 2.0 × 10^5^ years) and ^90^Sr (*t*_1/2_ = 28.1 years) were sufficiently long compared to the duration of the column tests, no additional radioactive decay correction was applied to determine the final activity in the effluent samples.

### Diffusion experiment

**Fig. 4:**
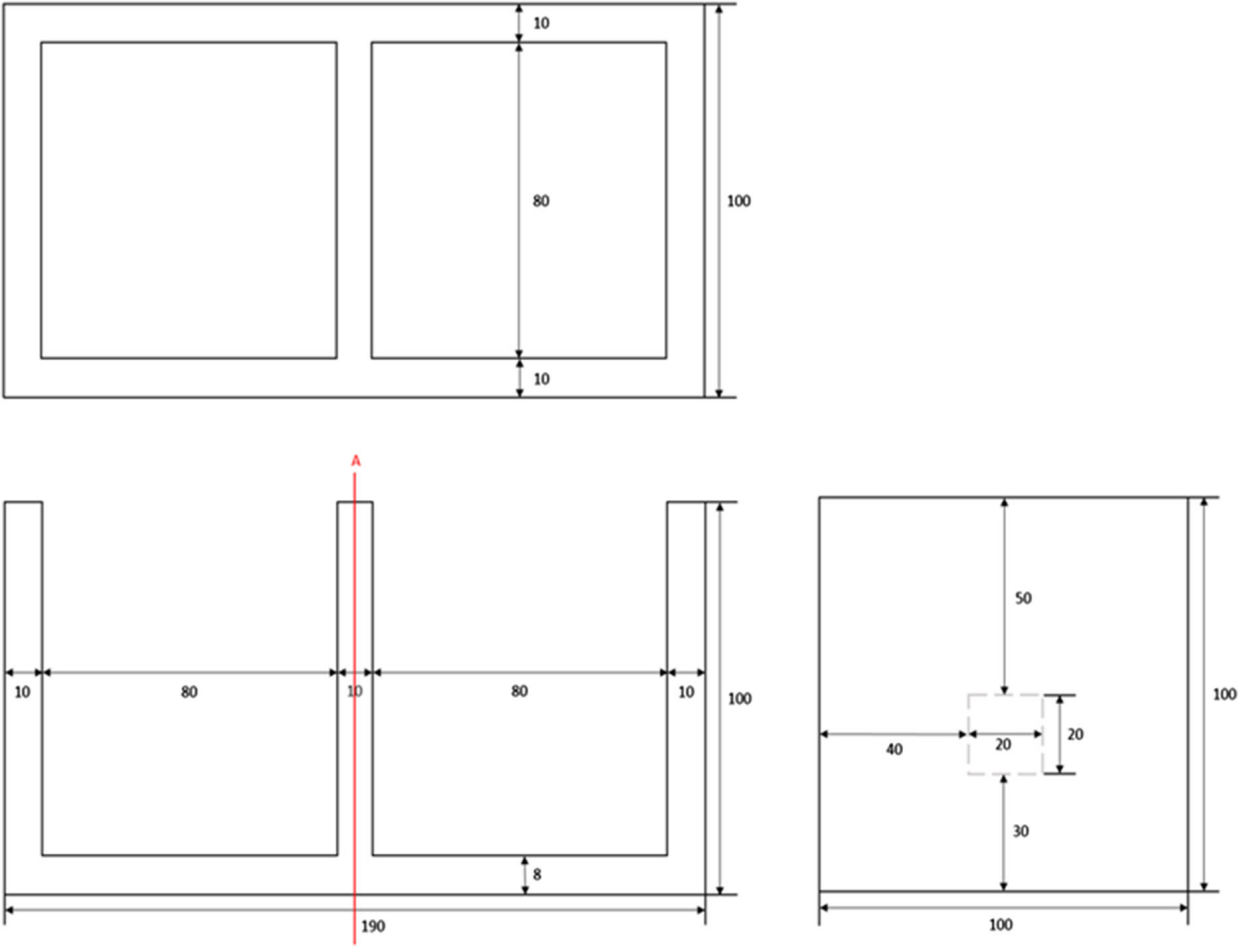
The schematic diagram of diffusion cell (unit, mm)

Diffusion experiments of ^3^H, ^99^Tc, and ^90^Sr through intact samples were carried out by the through-diffusion method at 25 °C under ambient conditions. The diffusion experiment equipment is shown in Fig. 2c. An acrylic diffusion cell which consists of two cells, tracer and measurement cell, was used in the experiments. The schematic diagram of cells is shown in Fig. 4. The inner size of each cell is 80 mm × 80 mm × 92 mm (length × width × height) and volume of each cell is 500 mL. A rock sample was placed between these cells. The rock sample was fixed in the hole with silicon glue. The properties of rock sample were determined before diffusion experiments started. The size of rock sample was 20 mm × 20 mm × 5 mm (length × width × height). In order to investigate the filling/coating materials of fractured rock sample, with and without filling/coating material, sample specimen was installed in diffusion cells. Before the diffusion experiments started, each diffusion cell was tested for leaks and the diffusion specimen in the cell was saturated for at least 7 days with the same background solution, which was for the experiments. After full saturation, solutions in both tracer and measurement cells were decanted carefully. For radionuclide diffusion experiments, radionuclide solution was poured into the tracer cell after completing the Br measurement. Solution in both tracer and measurement cells were fully mixed by magnetic stirrers. The pH of solutions was controlled at 7. The pH values of the solutions in both cells were measured and adjusted if necessary. Samples were collected periodically from both measurement and tracer diffusion cells. After sampling, an identical volume of DIW was added to the measurement cell to keep the solution volume constant. Duplicate samples were collected and mean value was used. The experiments were continued for 55 days for ^3^H, 64 days for ^99^Tc, and 228 days for ^90^Sr. The concentration of diffused solute through the cross-sectional area was measured from the measurement cell and plotted as a function of time. The effective diffusion coefficient of the radionuclide was determined using the time-lag method (Crank 1975; Shackelford 1991).

In this study, the time-lag method was used to obtain the diffusion coefficient, and it has been used to obtain the permeability constant, and the solubility of a gas flowing through a porous membrane (Crank 1975; Shackelford 1991). The diffused substance through a specimen with a constant concentration in the tracer cell, *C*_1_ at *x* = 0 and a very small concentration in the measurement cell, *C*_2_ (*C*_2_ ≪ *C*_1_) at *x* = *X* is (Crank 1975):

3 $$\frac{Q}{{X{C_1}}} = \frac{{{D_{\text{eff}}}t}}{X^2} - \frac{\alpha }{6} - \frac{2\alpha }{\pi^2}\mathop \sum \limits_{n = 1}^\infty \frac{{x{{\left( { - 1} \right)}^n}}}{n^2}\exp \left( { - \frac{{{D_{\text{eff}}}{n^2}{\pi^2}t}}{{{X^2}\alpha }}} \right)$$

where *Q* = total amount of diffusing component passed through the sample slab per time (M/L^2^), *X* = thickness of a sample (L), *C*_1_ = concentration at the tracer cell (*C*_1_ ≫ *C*_2_) (M/L^3^), *C*_2_ = concentration at the measurement cell (M/L^3^), and *D*_eff_ = effective diffusion coefficient for non-reactive solute (L^2^/t). This is replaced by *D*_app_ (apparent diffusion coefficient) for a reactive solute. Where *t* = time (t), α = rock capacity factor (= θ_tot_ + K_d_ρ_b_), *θ* = porosity, and *ρ*_b_ = bulk density. As time (*t*) approaches in infinity, the exponential term in Eq. 3 is negligible and Eq. 3 becomes

4 $$Q = {\text{\;}}\frac{{{C_1}{D_{\text{eff}}}}}{X}t - {\text{\;}}\frac{{{C_1}X\alpha }}{6}$$

with a slope, $\frac{{{C_1}{D_{\text{eff}}}}}{X}$ and an intercept on the time axis, $\frac{{{C_1}X\alpha }}{6}$. The slope and the intercept on the time axis of the extrapolating liner equation give the diffusion coefficient and rock capacity factor, respectively.

In this study, the concentration of radionuclide in the measurement cell was measured as a function of time and *D*_eff_ was obtained from the slope of the linear equation. The tortuosity was determined using the relation between molecular diffusivity and effective diffusivity, and the calculated tortuosity was also used to determine the *D*_eff_ and *D*_app_. The retardation factor, *R* was determined using the distribution coefficient obtained from the linear isotherm in the batch experiments. Previously measured bulk density and porosity for each sample slab were also used to determine the retardation factor.

## Results and discussion

### Characteristics of fractured rock

**Fig. 5:**
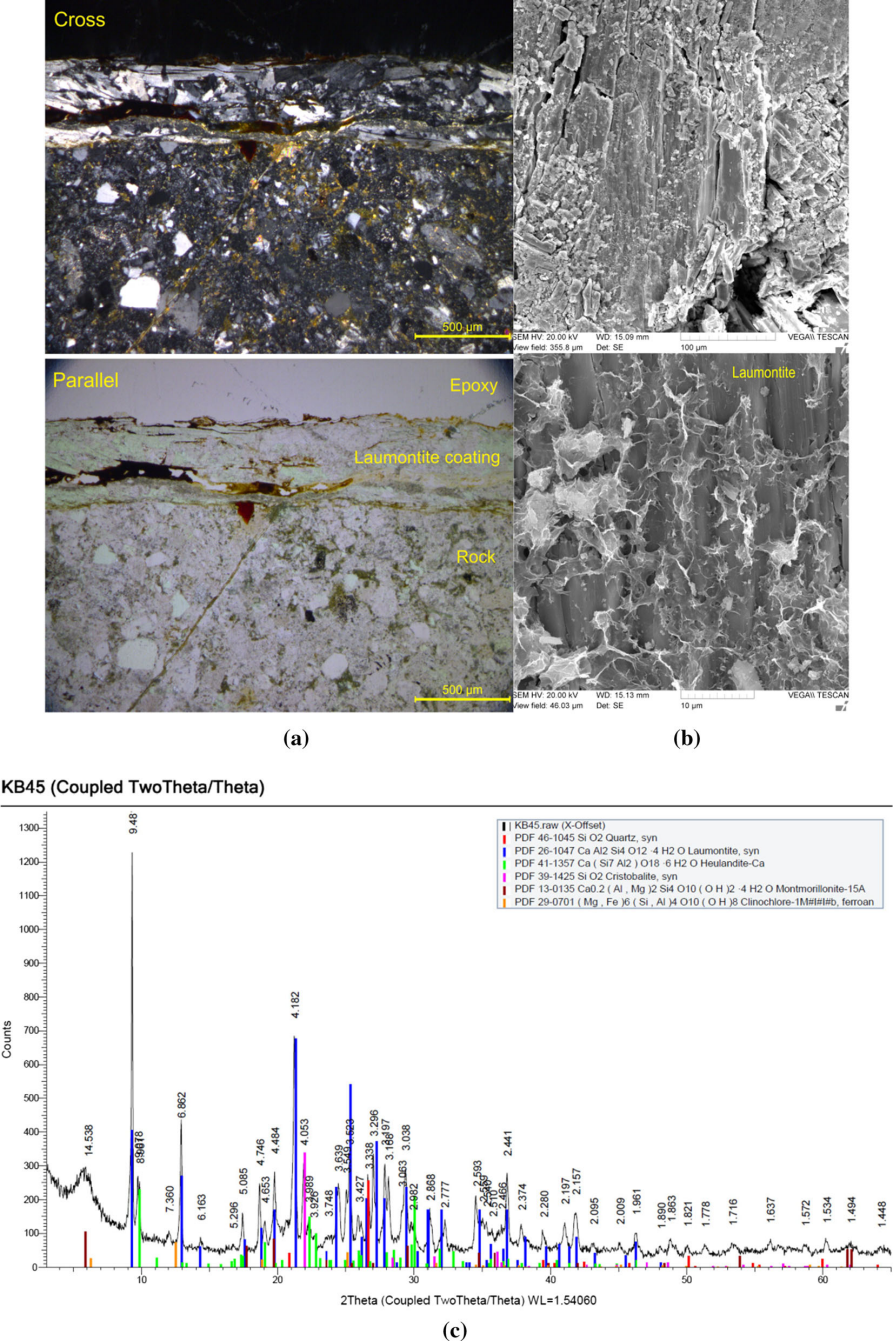
**a** PLM, **b** SEM, **c** XRD result of fractured rock

**Fig. 6:**
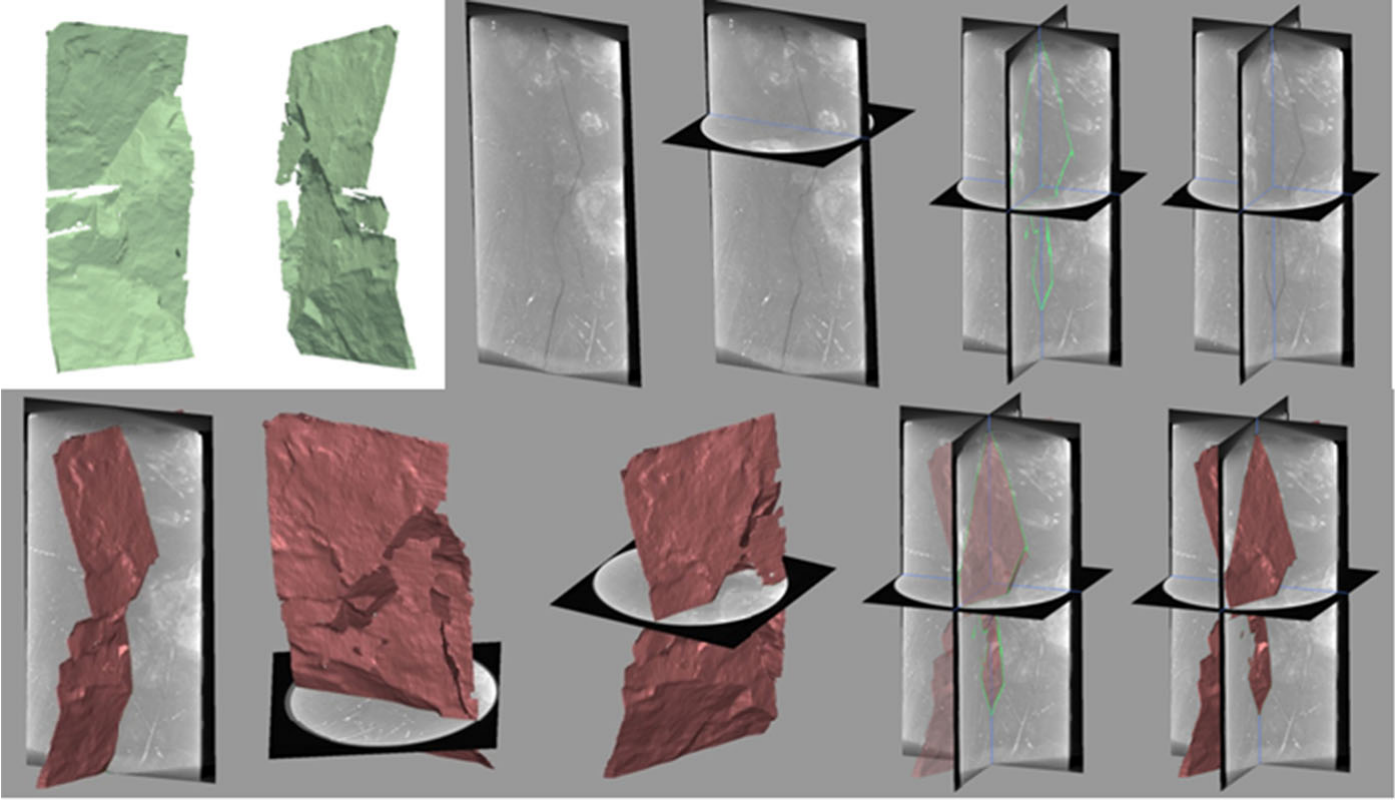
Result of XMT for core sample with fractures

The yellowish-brown filling/coating material of fractured rock sample was investigated by PLM, SEM, and XRD. The results of PLM, SEM, and XRD are shown in Fig. 5. The average thickness of filling/coating material is 500 μm, and zeolite was coated in the surface of rock. The filling/coating material was analyzed by XRD with reference intensity ratio method, and the main mineral is heulandite (32%) and laumonite (55%). The fractured rock sample for column experiment. The XMT can visualize the spatially heterogeneous surface of fractured rock sample. To visualize the formation, growth, sealing of internal fractures, and the fracture connectivity of fractured rock, the XMT was used. The visualization of fractured rock sample for column experiment is shown in Fig. 6. It shows the fractured main pathway which is zigzag passage, and the surface of fractured rock which is uneven. In addition, the computational fluid dynamics simulation was performed to get the volume of fracture (1.38 × 10^−6^ m^3^), and the permeability (2.54 × 10^−9^ m^2^).

### Batch sorption experiments

The equilibrium partition constant, generally referred to as a *K*_d_ (distribution coefficient), has been used to quantify sorption that is kinetically fast and reversible. The distribution coefficient results of ^99^Tc and ^90^Sr with fractured filling/coating material using groundwater are shown in Tables 1 and 2. The sorption results of ^3^H were not shown because the initial concentration (*C*_0_) and measured concentration (*C*_q_) of tritium are same in the experiment, the *K*_d_ value become zero in both with and without fracture-filling/coating materials. It means that tritium has no sorption characteristic.

**Table 1:** Distribution coefficient of ^90^Sr (w/: with, w/o: without)

^90^Sr	*K*_d_ (mL/g)	Average *K*_d_ (mL/g)	*K*_d_ SD
w/fracture-filling rock 1	48.178		
w/fracture-filling rock 2	44.704	45.1	2.919
w/fracture-filling rock 3	42.376		
w/o fracture-filling rock 1	30.486		
w/o fracture-filling rock 2	29.817	30.3	0.449
w/o fracture-filling rock 3	30.671

**Table 2:** Distribution coefficient of ^99^Tc (w/: with, w/o: without)

^99^Tc	*K*_d_ (mL/g)	Average *K*_d_ (mL/g)	*K*_d_ SD
w/fracture-filling rock 1	0.535		
w/fracture-filling rock 2	1.258	0.9	0.364
w/fracture-filling rock 3	0.965		
w/o fracture-filling rock 1	1.445		
w/o fracture-filling rock 2	0.458	1.1	0.553
w/o fracture-filling rock 3	1.384		

After experiments involving batch sorption, distribution coefficients (*K*_d_) of the radionuclides were obtained, and sorption properties for each radionuclide were analyzed. Increasing *K*_d_ values were observed in the sequence ^90^Sr, ^99^Tc, and ^3^H regardless of the geological media tested. The *K*_d_ of ^90^Sr (45.1 mL/g) with fracture-filling/coating materials is higher than without fracture-filling/coating materials (30.3 mL/g). In case of ^99^Tc, there are similar *K*_d_ in both with and without fracture-filling/coating materials (0.9 and 1.1 mL/g). For all sorbing radionuclides, *K*_d_ values for the fracture-filling/coating material were observed to be higher than those for without fracture-filling/coating material regardless of the groundwater. These higher *K*_d_ values are the result of zeolite mineral in filling/coating material of fractured rock. The mineralogical composition of the solid materials was observed to be important in the sorption of sorbing radionuclides. In this study, sorption distribution coefficient (*K*_d_) of radionuclide was determined and used to increase our understanding of radionuclide retardation through fracture-filling/coating materials. Transport behavior of radionuclide was affected by geological environment. The physical/chemical characteristic and ion-exchange of rock surface is a main cause to adsorption capacity, and the geological materials of rock surface are also important cause to adsorption coefficient (Andersson et al. 1983). Previous study shows that heulandite and laumontite affect to adsorption of radionuclide, and especially uranium and strontium was strongly affected by heulandite and laumontite in geological environment, so the retardation of radionuclide was increase (Chernjatakaja 1988; Matijašević et al. 2006; Baik et al. 2009). These results show that the released radionuclide from radioactive waste repository can be transferred through fractured rock and it can be retarded by fractured filling/coating materials. The strontium, which has high adsorption capacity, is retarded in fractured rock with heulandite and laumontite compared with technetium and tritium.

### Flow-through column experiments

**Fig. 7:**
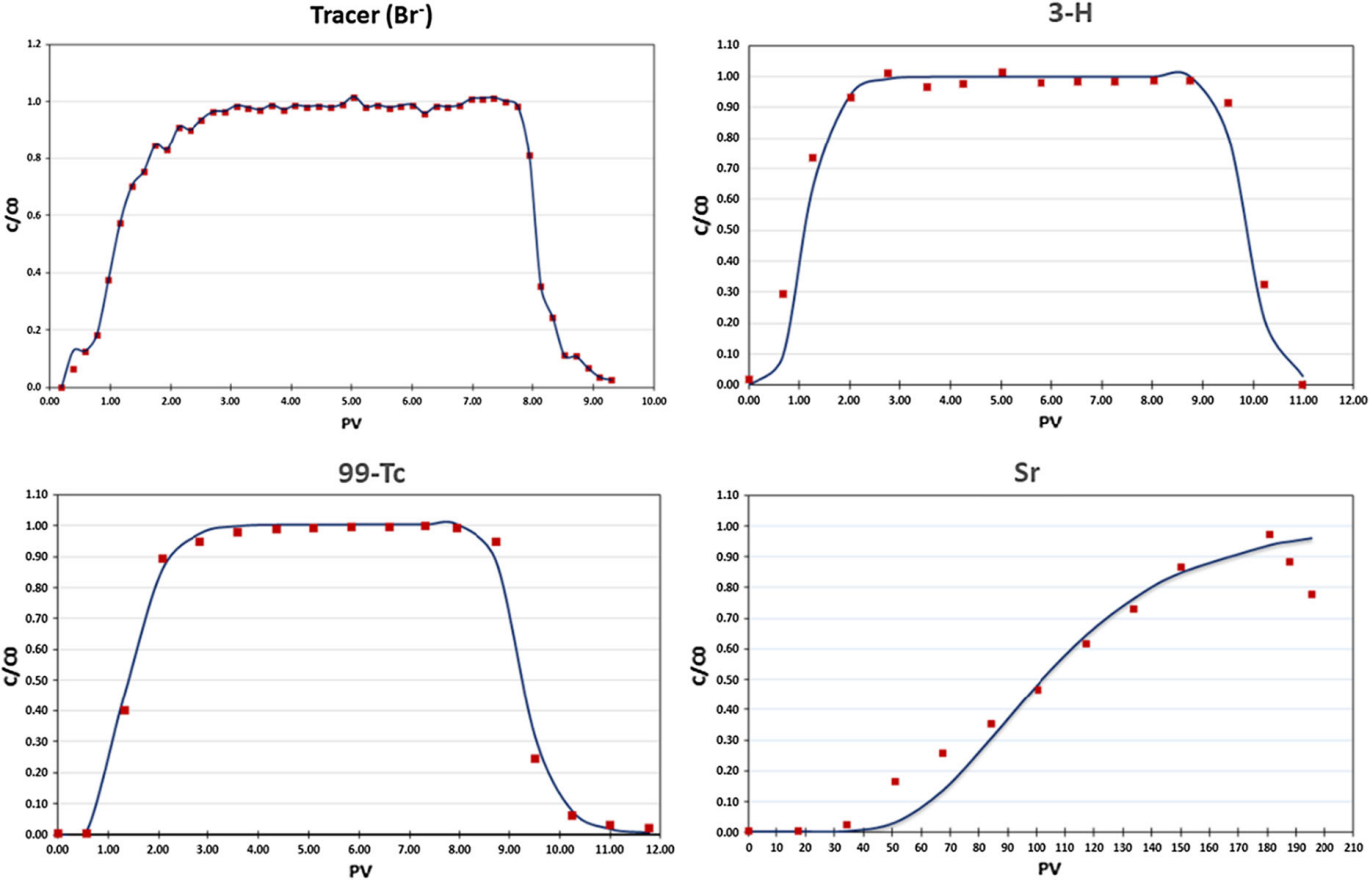
Breakthrough curves for Br and radionuclide and CXTFIT model fit

The migration of ^3^H, ^99^Tc, and Sr spiked into LILW repository groundwater through fractured rock near repository was also evaluated using flow-through column experiments in unsaturated condition. Breakthrough curves for Br and radionuclide are presented in Fig. 7. Dispersion coefficient (*D*) values determined from Br breakthrough curve fits are very similar *D* values, which indicated that all column experiments were well prepared and conducted under similar flow environments whit no significantly varying permeability and porosities. Radionuclide breakthrough curves were analyzed with either the classical equilibrium advection–dispersion equation or non-equilibrium two-region (mobile–immobile) modeling. The column conditions and transport parameters obtained from the column experiments are shown in Table 3.

**Table 3:** Column conditions and transport parameters

Column	pH	*V* (cm/min)	*D* (cm^2^/min)	*R*	*K*_d_ (cm^3^/g)	*r* ^*2*^
^3^H	6.8	0.2071	0.2097	1.2	0.022	0.961
^99^Tc	6.8	0.2071	0.2097	1.495	0.05	0.995
Sr	6.8	0.2071	0.2097	109.8	12	0.949

When the fractured rock column was contacted with radionuclide spiked groundwater, radionuclide transport was retarded by the fractured rock column due to the sorption affinity of radionuclide on the surface of fractured rock sample under groundwater in unsaturated condition. Sr transport was more considerably retarded by the column due to the high sorption affinity of Sr on the fractured rock sample than ^3^H and ^99^Tc. Therefore, Sr transport behavior through fractured rock under groundwater condition was significantly retarded compared with that of ^3^H and ^99^Tc. The breakthrough curves for Br, ^3^H, ^99^Tc, and Sr with CXTFIT model fit are presented in Fig. 7. The transport behavior of ^3^H and ^99^Tc was identical to that of the non-reactive tracer (Br). Measured transport parameters indicated no chemical interaction of ^3^H, ^99^Tc on the LILW fractured rock, and the K_d_ value calculated from column experiments was different to that of the batch sorption experiments. Different *K*_d_ values between batch and column experiments were due to the different experimental conditions, as found in other previous studies. A classical equilibrium model within the CXTFIT code showed reasonable fit results (*r*^*2*^ = 0.949–0.995) for ^3^H, ^99^Tc, and Sr breakthrough curves. The breakthrough of ^3^H and ^99^Tc shows earlier than that of Sr, which shows a small value of *R* < 1.0 (*R* of ^3^H = 1.2, and *R* of ^99^Tc = 1.495), and R of Sr is 109.8. Due to the slight sorption affinity of ^99^Tc on the fractured rock under groundwater conditions, ^99^Tc transport behavior through the fractured rock was slightly retarded compared with that of the non-reactive tracer (Br), and ^3^H. Because of the intermediate and high sorption *K*_d_ values determined for Sr from the batch sorption experiments, the flow rates used in the column experiments for Sr was increased over column experiments. Based on sorption and column results for Sr through LILW fractured rock, Sr is considered to show an intermediate amount of retardation, irrespective of being present as an anionic species in the LILW site subsurface environment. Although a fast flow rate was used for Sr column experiment, a breakthrough curve was not completely obtained even after 200 pore volumes. However, the observed relative effluent concentrations for Sr transport through LILW fractured rock in unsaturated condition are highly retarded. As the result of the batch sorption experiments, Sr has a high retardation value (109.8) calculated from the column results through LILW fractured rock on unsaturated conditions.

### Diffusion experiments

**Fig. 8:**
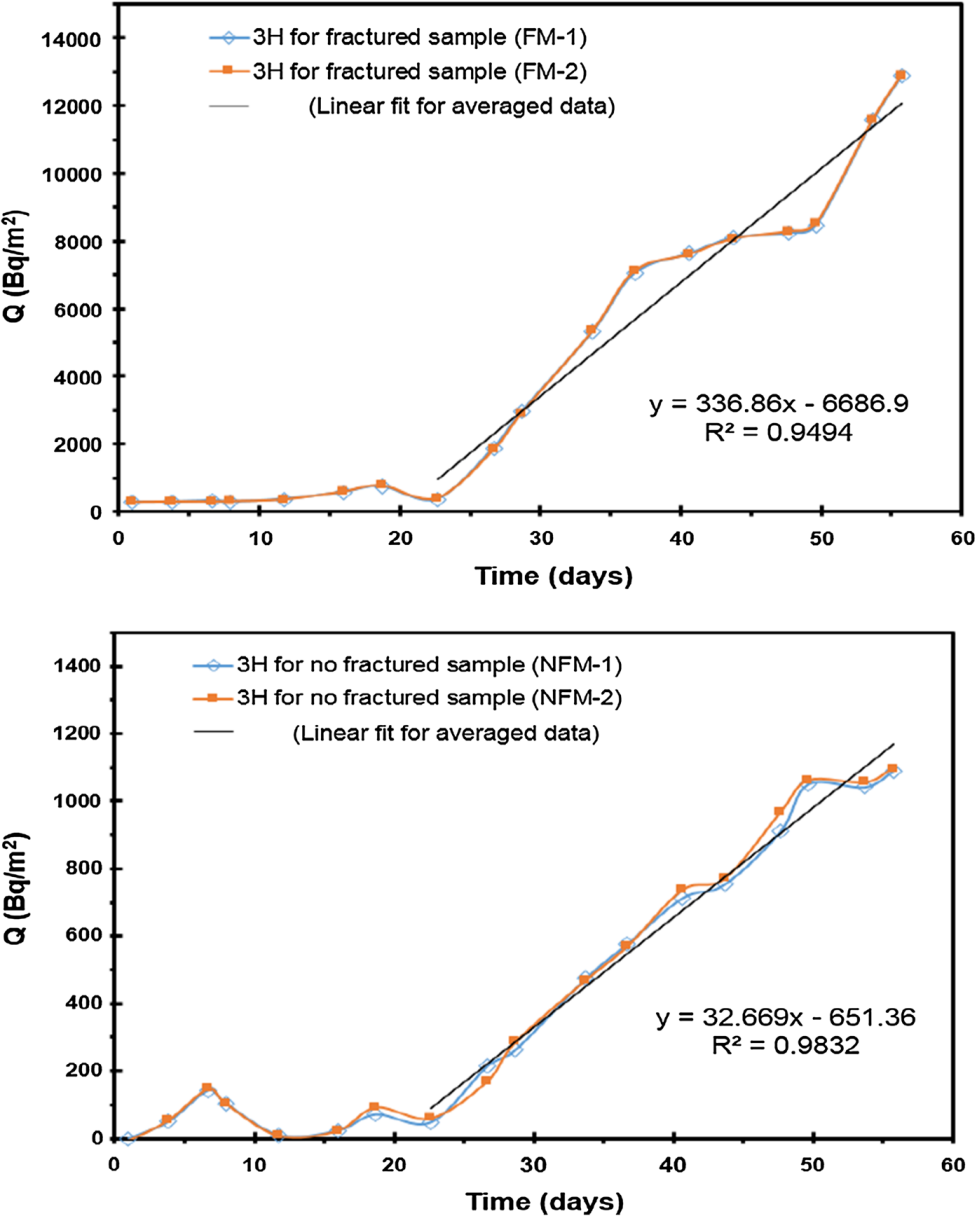
Experimental results and linear regression line for ^3^H diffusion through with and without fractured sample

**Fig. 9:**
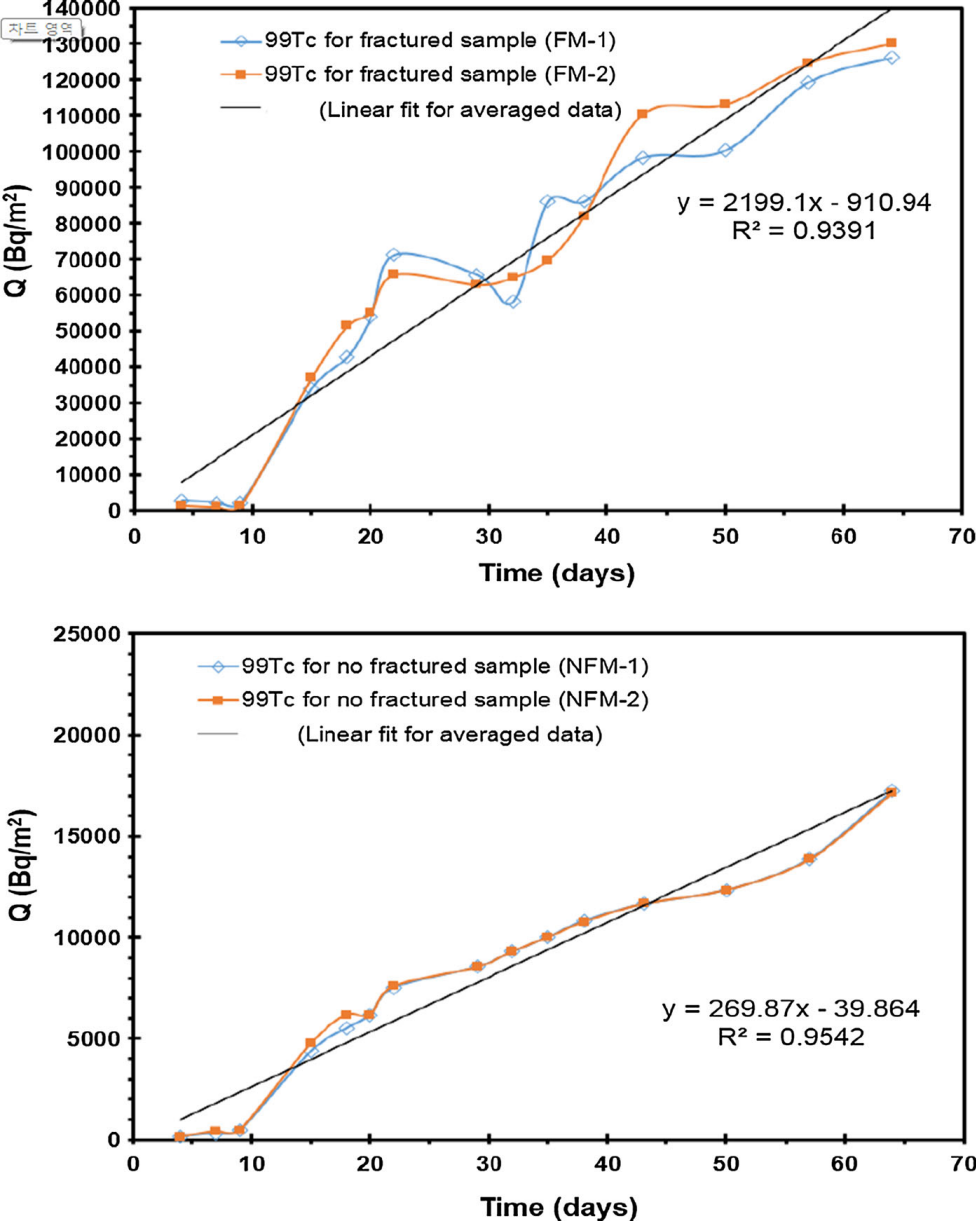
Experimental results and linear regression line for ^99^Tc diffusion through with and without fractured sample

**Fig. 10:**
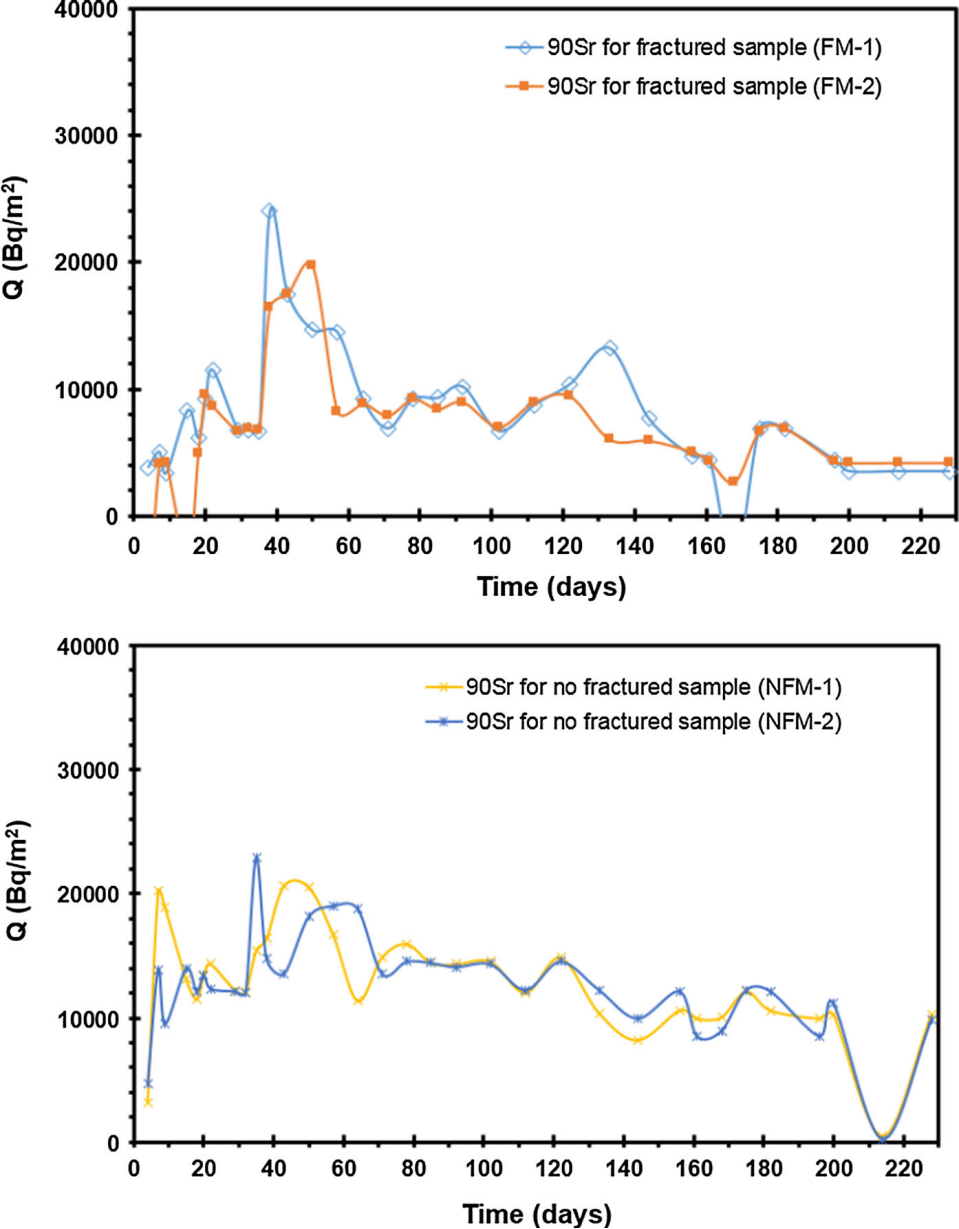
Experimental results and linear regression line for ^90^Sr diffusion through with and without fractured sample

The total amount of ions diffused, ^3^H, ^99^Tc, and ^90^Sr, through the fractured rock specimen with and without filling/coating material was plotted as a function of time to determine the *D*_eff_. The diffusion of ^3^H, ^99^Tc, and ^90^Sr through the fractured rock specimen with and without filling/coating material is, respectively, shown in Figs. 8, 9, and 10. The breakthrough obtained from ^3^H and ^99^Tc diffusion experiment shows a curve in transient state during the early short period and a straight line in steady state after a certain amount of time has passed. The amount of radionuclide diffused through specimen was measured in the measurement cell as a function of time. *D*_eff_ of ^3^H and ^99^Tc was estimated using the slope in the equation of linear regression line. However, *D*_eff_ of ^90^Sr was estimated by different method due to strong sorption affinity. Low concentration of Sr was detected in the measurement cell. In addition, a continuous decrease in Sr concentration was found in the tracer cell. Although ^3^H, ^99^Tc concentration in the tracer cell also decreased as time increased, the decrease in concentration of ^3^H, ^99^Tc was minor and the ^3^H, ^99^Tc concentration in the tracer cell was still much higher than that of the measurement cell. However, ^90^Sr concentration decreased to less than 50% of initial concentration within 2 days. Even though the background concentration increased, the diffusion experiments of this study did not show any discernable amounts of ^90^Sr in the measurement cell.

The amount of ^90^Sr as a function of time in the tracer cell is shown in Fig. 7. The concentration decrease in the tracer cell and the absence of ^90^Sr concentration in the measurement cell result from sorption affinity of ^90^Sr on specimen with and without coating material on the surface of fractured rock. Even though ^90^Sr can diffuse inside pores of the specimen, due to strong sorption affinity, ^90^Sr ions are considered to be stuck to the surfaces of pores in the specimen. Therefore, ^90^Sr could not be found in the measurement cell after passing through specimen. Because no ^90^Sr was found in the measurement cell and the concentration of ^90^Sr decreases in the trace cell, the time-lag method could not be used to determine the diffusivities of ^90^Sr. Instead, *D*_app_ of ^90^Sr through specimen is determined using Eq. 5.

5 $${D_{\text{app}}} = \;\frac{{{D_{\text{mol}}}}}{{\left( {{\text{retardation}}\;{\text{factor}}} \right)\left( {{\text{rock}}\;{\text{factor}}} \right)}}$$

*D*_mol_ of ^90^Sr was calculated using the Nernst-Haskell equation (Reid et al. 1987).

*D*_eff_ and *D*_app_ are shown in Table 4. The effective diffusion coefficients for ^3^H with and without fractured sample showed similar results. The results of slight differences between *D*_eff_ of ^3^H through fractured sample with and without coating material in surface of specimen are considered to result from the different characteristic of coating material in surface of specimen. The effective diffusion coefficients for ^99^Tc also showed similar results with and without fractured sample, and the slight differences of *D*_eff_ of ^99^Tc are considered to same reason. The apparent diffusivity of ^90^Sr with fractured sample is little bit higher than without fractured sample. Because the affinity of ^90^Sr to fracture-filling/coating material is high, the more amount of ^90^Sr is hocked inside of diffusion specimen with filling/coating material than without filling/coating material. So the diffusion amount of ^90^Sr is higher through specimen with filling/coating material. To do think in a different way, because the holding amount of ^90^Sr in filling/coating material is high, the transport of ^90^Sr is retarded.

**Table 4:** The diffusivity of radionuclide through specimen

(m^2^/sec)	*D*_eff_ of ^3^H	*D*_eff_ of ^99^Tc	*D*_app_ of ^90^Sr
With fracture-filling specimen	2.399E−11	1.86E−11	4.73E−15
Without fracture-filling specimen	2.589E−12	2.56E−12	1.23E−15

## Conclusions

In this study, we measured *K*_d_ values and investigated sorption characteristics of ^3^H, ^99^Tc, and ^90^Sr for fractured rock in unsaturated zone at the Wolsong LILW repository. The main materials of filling/coating materials in fractured rock near the Wolsong LILW disposal facility is zeolite (heulandite (87%) and laumontite (55%)). The sorption characteristic of ^90^Sr is stronger than ^99^Tc therefore, radionuclides such as ^90^Sr are thought to bind even stronger than ^99^Tc and ^3^H. The *K*_d_ values for ^90^Sr were found to be at intermediate levels, however, *K*_d_ values for ^99^Tc were very low, and *K*_d_ values for ^3^H cannot be calculated. In addition, when fracture-filling materials exist, distribution coefficient of ^90^Sr is higher than without fracture-filling materials because of zeolite mineral in fractured rock. However, the *K*_d_ value of ^99^Tc is similar in both with and without fracture-filling/coating materials. This study shows that the mineralogical composition of the geological media is an important factor when sorbing radionuclides, especially for strongly sorbing radionuclides. The transport of radionuclide in LILW site through fractured rock can be retarded by filling/coating materials. Specifically, ^90^Sr which have intermediate adsorption characteristic transport through fracture rock was retarded. This work involved a geochemical characterization of the filling/coating material in fractured rock near Wolsong LILW repository. The results of adsorption, diffusion, and flow-through column experiments were provided the basic information and data for the safety assessment of LILW repository. Based on results obtained from batch experiments, ^90^Sr bind strongly on fractured rock samples. Due to the strong sorption affinities, calculated apparent diffusion coefficients for ^90^Sr was high, and the diffusivity of ^90^Sr through specimen with fracture-filling/coating material was higher than without fracture-filling/coating material. Sorption and diffusion are important retardation processes for radionuclide migration when radionuclides are released from a radioactive waste repository. Thus, sorption and diffusion experimental data for radionuclides, which usually are represented as distribution coefficients and diffusion coefficients, are needed for the safety assessment of a radioactive waste repository. In this study, the sorption and diffusion results of radionuclide in fractured rock under unsaturated condition give the important information in repository, and support the understanding about retardation of radionuclide through fracture rock.
